# The role of chronic disease in the disparity of influenza incidence and severity between indigenous and non-indigenous Australian peoples during the 2009 influenza pandemic

**DOI:** 10.1186/s12889-022-12841-6

**Published:** 2022-07-05

**Authors:** Rashmi Dixit, Fleur Webster, Robert Booy, Robert Menzies

**Affiliations:** 1 Westmead Clinical School, University of Sydney, NSW 2145 Sydney, Australia; 2 School of Public Health and Community Medicine, University of New South Wales, NSW 2052 Sydney, Australia; 3 Westmead Institute for Medical Research, NSW 2145 Sydney, Australia; 4 Kirby Institute, Faculty of Medicine, University of New South Wales, NSW 2052 Sydney, Australia

## Abstract

**Background:**

The 2009 H1N1 influenza pandemic (influenza A(H1N1)pdm09) disproportionately impacted Indigenous peoples. Indigenous Australians are also affected by a health gap in chronic disease prevalence. We hypothesised that the disparity in influenza incidence and severity was accounted for by higher chronic disease prevalence.

**Methods:**

We analysed influenza data from Western Australia, South Australia, the Northern Territory, and Queensland. We calculated population prevalence of chronic diseases in Indigenous and non-Indigenous Australian populations using nationally-collected health survey data. We compared influenza case notifications, hospitalisations, intensive care admissions, and deaths reported amongst the total population of Indigenous and non-Indigenous Australians ≥ 15 years. We accessed age-specific influenza data reported to the Australian Department of Health during the 2009 ‘swine flu’ pandemic, stratified by Indigenous status and the presence of one of five chronic conditions: chronic lower respiratory conditions, diabetes mellitus, obesity, renal disease, and cardiac disease. We calculated age-standardised Indigenous: non-Indigenous rate ratios and confidence intervals.

**Findings:**

Chronic diseases were more prevalent in Indigenous Australians. Rates of influenza diagnoses were higher in Indigenous Australians and more frequent across all indices of severity. In those with chronic conditions, Indigenous: non-Indigenous influenza notification rate ratios were no lower than in the total population; in many instances they were higher. Rate ratios remained above 1·0 at all levels of severity. However, once infected (reflected in notifications), there was no evidence of a further increase in risk of severe outcomes (hospitalisations, ICU admissions, deaths) amongst Indigenous Australians compared to non-Indigenous Australians with a chronic disease.

**Interpretation:**

Higher rates of influenza infection was observed amongst those Indigenous compared to non-Indigenous Australians, and this difference was preserved amongst those with a chronic condition. However, there was no further increase in prevalence of more severe influenza outcomes amongst Indigenous Australians with a chronic condition. This suggests that the prevalence of chronic disease, rather than Indigenous status, affected influenza severity. Other factors may be important, including presence of multiple morbidities, as well as social and cultural determinants of health.

**Supplementary Information:**

The online version contains supplementary material available at 10.1186/s12889-022-12841-6.

## Introduction

The 2009 H1N1 influenza pandemic (influenza A(H1N1)pdm09) disproportionately impacted Indigenous populations in colonised countries, including Aboriginal and Torres Strait Islander peoples (Indigenous Australians) [1–7]. Colonised Indigenous populations around the world have a greater burden of chronic, non-communicable diseases compared to their non-Indigenous counterparts [2, 8–11]. Both seasonal and pandemic influenza affect those with chronic diseases more frequently and severely [3, 7, 12–15]. It has frequently been hypothesised that the greater influenza A(H1N1)pdm09 disease burden and severity in Indigenous populations is due to their larger burden of chronic diseases, but very few studies have directly examined this [3, 16].

While the 2009 pandemic strain appeared over 10 years ago, the question is still relevant. Influenza A(H1N1)pdm09 continues to be predominately circulate, as indicated by an World Health Organization report that showed it represented 49% of strains in 2019 [17]. Moreover, emergent strains with pandemic potential have the capacity to cause greater disease severity amongst First Peoples populations [18]. The nature of interactions between acute influenza, Indigeneity, and chronic diseases during the 2009 pandemic are also likely to apply to other seasonal influenza strains. Other reports have been based on data collected from states or / regions rather than national data, and have not primarily examined the relationship between differential chronic disease rates between Indigenous and non-Indigenous patients and 2009 pandemic influenza severity. We accessed national data sets with a view to specifically analyse these associations. The current COVID-19 outbreak has likewise provided an opportunity for researchers to study the impact of viral pandemics on Indigenous populations in colonised nations, with respect to background health and socioeconomic inequities, with a recognition of the urgency to collect prospective data [19].

Our central tenet is: if higher rates of pandemic influenza infection (notifications) and influenza severity in Indigenous compared to non-Indigenous peoples were due to the higher prevalence of chronic noncommunicable diseases, then this disparity would be eliminated by only comparing influenza rates amongst those with chronic conditions. However, if other factors are also influential, then disparities in disease and severe outcomes between Indigenous and non-Indigenous people would remain after restricting the analysis to those with chronic diseases.

Acute influenza surveillance was conducted nationally in Australia in 2009. National influenza notification rates reflect the incidence of influenza infection; while hospitalisation, intensive care, and mortality data broadly reflect the incidence of progressively more severe outcomes, notwithstanding the limitations of these sources. Routine data collection provided Indigenous and chronic disease status on individual cases.

The aim of this study was to determine whether the higher incidence of infection and severity of influenza A(H1N1)pdm09 in Indigenous populations compared to the non-Indigenous Australian population was reduced or eliminated after stratifying by chronic disease prevalence.

## Methods

### Population prevalence of chronic diseases

Prevalence data for chronic conditions amongst Australians were obtained by analysing the Confidentialised Unit Record Files of Health Surveys conducted by the Australian Bureau of Statistics (ABS). For Indigenous Australians, data were from the 2012–13 National Aboriginal and Torres Strait Islander Health Survey, collected between April 2012 and February 2013 from 5000 private dwellings across Australia [20]. Prevalence data for non-Indigenous Australians were from the 2011–12 National Health Survey, conducted between 6 March 2011 and 17 March 2012 from a sample of 15,500 private dwellings across Australia [21]. Whilst the 2011-12 National Health Survey included data on all Australians regardless of ethnicity, as Indigenous Australians make up only 3.3% of the total population, the results were used to reflect the prevalence of chronic conditions amongst the 96.7% of Australians that are non-Indigenous [22]. Only data for those aged ≥ 15 years were available from each survey, therefore our analysis was limited to these age groups. Within the health surveys, all chronic disease data were self-reported except obesity, which was directly measured. Respondents were asked whether they had been identified with the condition, and whether the condition was current and long-term; and were classified as having a chronic disease for the purposes of our study if they answered positively to both questions. All reported long-term medical conditions were coded to a classification developed for use in the ABS Health Surveys, based on an international disease classification system (see Additional file 1) [23].

Percentage prevalence of each condition in the morbidity, mortality, and health survey data was stratified by the following age groups for each state and territory: 15–24 years, 25–34 years, 35–44 years, 45–54 years, and ≥ 55 years. The survey percentages were adjusted using weightings for age, gender, and state or territory provided to the ABS, to adjust for differences between survey and census populations [24].

### Influenza laboratory notifications, hospitalisations, ICU admissions, and deaths

We obtained data on influenza cases during the 2009 ‘swine flu’ pandemic from the Communicable Diseases Network of Australia (CDNA), a division of the Department of Health and Aging (DoHA). Influenza surveillance data were actively collected during the pandemic, under the provisions specified within the National Health Agreement, and state or territory public health acts. Cases of laboratory-confirmed influenza A(H1N1)pdm09 infections, hospitalisations, ICU admissions, and deaths were collected by states or territories from general practitioners, hospitals, and laboratories and reported to the National Incident Room of the DoHA. The DoHA entered the data onto the NetEpi database, a web-based outbreak case reporting system. Data from those 15 years of age and older, were obtained from the CDNA, to allow NetEpi data to match the age range of people surveyed for chronic conditions. Data from 1 April 2009 to 31 December 2009 were examined. For the total (general) population, data were only available from all of Australia, not by state or territory (Table 5).

Indigenous status was recorded by self-reporting to health professionals. If a patient was clinically unable to answer questions (e.g., they were receiving ventilation or they had died) their next of kin was asked about the patient’s Indigenous status. ‘Non-Indigenous Australians’ included those for whom Indigenous status was recorded as ‘not Aboriginal or Torres Strait Islander peoples.’ Data in which Indigenous status was not specified were omitted from this analysis. The presence of chronic disease in a case was reported by the health care provider or obtained from clinical notes.

The exact definitions applied to the chronic conditions were not available from NetEpi, and may have differed from that of the ABS Health Surveys, specified above. The CDNA NetEpi database was unable to provide data on those cases of influenza without any chronic conditions. We could not calculate these simply by subtracting cases with a chronic condition, as a case may have had more than one chronic condition.

### Role of the funding source

There was no funding received for the purpose of this study.

#### Statistics

The analyses that were conducted were limited by data availability. Table 5: amongst those with chronic conditions, data on influenza notifications stratified by Indigenous status were available only from Western Australia (WA) and South Australia (SA); whereas data on hospitalisations, ICU admissions, and deaths from influenza stratified by Indigenous status were available from WA, SA, the Northern Territory (NT), and Queensland (QLD). This represents 57·5% of Aboriginal and Torres Strait Islander peoples within Australia. For comparison, the background population dubbed ‘all Australia’, those with and without chronic conditions, data on notifications and hospitalisation were available by state and territory; whereas, data for ICU admissions and death were only available for the whole of Australia, not separated by state or territory. We combined data from states or territories where equivalent data were available, to increase statistical power.

To obtain estimated population numbers for each chronic condition, we multiplied the percentage prevalence of each chronic condition reported within the Australian Bureau of Statistics (ABS) Health Surveys (Indigenous and all Australian, the latter serving as a proxy for non-Indigenous) for each age group by the total 2011 census population numbers [24]. Percentage prevalence of each condition in the health survey data provided to the Australian Bureau of Statistics were adjusted using weightings for age, gender, probability of being selected, non-response rates and state or territory to adjust for differences between survey and census populations [25]. Our rate ratios reflected weighted and age standardized calculations of disease prevalence and infection incidence.

For rates of influenza within the total population we used reported total cases of influenza for each state or territory as the numerator, and ABS Census population data as the denominator. For rates of influenza amongst those with each chronic condition, we used reported cases of influenza with the comorbid chronic disease within each state or territory as the numerator, and the estimated numbers of people with the chronic disease (as described above) for the denominator.

We calculated rates for influenza notifications, hospitalisations, ICU admissions, and deaths. These rates were then used to calculate Indigenous: non-Indigenous rate ratios (RRs) and 95% confidence intervals (CIs). We performed direct age-standardisation of the influenza case data with the non-Indigenous population as a reference. We calculated 95% CIs using the method described by Armitage and Berry [26]. We defined lack of statistical significance as overlapping CIs—a conservative indicator of statistical significance. While indirect age-standardisation is recommended for analyses of small numbers of events, that was not feasible in this study as it required replacement of those with chronic conditions as the denominator with a general reference population, which would have made it impossible to test our hypothesis. Confidence intervals were calculated using the Poisson process described by Liddell, via a statistical calculator [27].

Chronic disease population estimates had 95% CIs derived from the ABS Health Surveys. Therefore, influenza rates and rate ratios in chronic disease populations were calculated using both the upper and lower chronic disease population estimates. Confidence intervals for the rates and ratios incorporated the full range generated from the upper and lower population estimates.

Ethics approval was granted on 17 May 2017 from the Human Research Ethics Committee of the University of Sydney; Project no.: 2017/356.

## Results

### General

All chronic conditions analysed—chronic lower respiratory conditions, diabetes mellitus, obesity, renal disease, and cardiac disease—were more prevalent in Indigenous Australians (Table 1). Tobacco use amongst those diagnosed with influenza A(H1N1)pdm09 during the 2009 pandemic was not available for further analysis; however, tobacco underlies many respiratory diseases in adults, and so usage statistics have been included in Table 1.

**Table 1:** Age Adjusted Chronic disease prevalence in Aboriginal and Torres Strait Islander peoples vs. Total Population ^a^

	State/territory	Rates	RR^**b**^	95% CI^**c**^
**Chronic lower respiratory conditions**	WA/SA^d^	21.0% vs. 13.7%	1·53	1·36–1·73
	WA/SA/NT/QLD^e^	18.2% vs. 13.6%	1·34	1·23–1·46
	All Australia	30.0% vs. 13.4%	2·23	2·11–2·36
**Smoking**	WA/SA	44.0% vs. 19.1%	2·40	2·21–2·60
	WA/SA/NT/QLD	46.2% vs. 18.4%	2·41	2·28–2·55
	All Australia	43.3% vs.17.3%	2·50	2·39–2·61
**Diabetes mellitus**	WA/SA	18.9% vs. 5.5%	3·40	2·91–3·98
	WA/SA/NT/QLD	16.9% vs. 5.0%	3.36	2·98–3·75
	All Australia	21.6% vs. 5.4%	4·04	3·73–4·38
**Obesity**	WA/SA	4.9% vs. 2.9%	2.40	1.69-4.05
	WA/SA/NT/QLD	6.4% vs. 3.4%	1.98	1.86-2.82
	All Australia	8.5% vs. 3.5%	2·45	2·13–2·82
**Renal disease**	WA/SA	2.8% vs. 1.7%	1.67	1·16–2.39
	WA/SA/NT/QLD	2.7% vs. 1.4%	1.99	1·52–2.60
	All Australia	4.5% vs. 1.0%	4·43	3·66–5·35
**Cardiac disease**	WA/SA	10.1% vs. 4.7%	1·99	1·52–2·60
	WA/SA/NT/QLD	7.4% vs. 4.4%	2.17	1·78–2.64
	All Australia	10.2% vs. 4.8%	2·13	1·92–2·36

^a^ ≥ 15 years of age

^b^Rate ratio

^c^Confidence interval

^d^Western Australia / South Australia

^e^Western Australia / South Australia / the Northern Territory / Queensland

Amongst the total population (all notified cases of influenza with no reference to chronic disease status) including all ages, Indigenous status was most complete in WA and SA (81.4% Indigenous status reported), with data less reliable from WA/SA/NT/QLD (68.7%) and from the whole of Australia (60.7%) (Table 2). Amongst those with influenza and any of the chronic diseases, Indigenous status was again most complete from WA/SA, and less so when the four states were combined (Table 3). As stated above, cases with missing Indigenous status were excluded from further analysis.

**Table 2:** Indigenous status distribution by state or territory—influenza notifications, total population^a^

	Indigenous	Non-Indigenous	Indigenous status not specified
**WA/SA**^**b**^	6·4%	75·0%	18·6%
**WA/SA/NT/QLD**^**c**^	13·9%	54·8%	31·3%
**All Australia**	10·8%	49·9%	39·3%

^a^With or without chronic disease

^b^Western Australia / South Australia

^c^Western Australia / South Australia / the Northern Territory / Queensland

**Table 3:** Indigenous status distribution by state or territory—influenza cases with chronic disease

	Indigenous status	Notifications	Hospitalisations	ICU admissions	Deaths
**WA/SA**^**a**^	Indigenous	13·7%	18·2%	··	··
	Non-Indigenous	82·1%	80·7%	··	··
	Indigenous status not specified	4·3%	1·1%	··	··
**WA/SA/NT/QLD**^**b**^	Indigenous	··	30·1%	26·7%	20·5%
	Non-Indigenous	··	41·3%	44·1%	44·6%
	Indigenous status not specified	··	28·6%	29·1%	34·9%

^a^Western Australia / South Australia

^b^Western Australia / South Australia / the Northern Territory / Queensland

### Rate ratios for indigenous compared to non-indigenous for influenza in the all Australia (total) population vs. chronic disease populations

Total numbers of influenza cases are presented in Table 4 by state or territory, severity, and chronic disease status, as well as their respective populations.

**Table 4:** Influenza cases for the total population and for each chronic condition^a^

	State/territory	Category	Indigenous Australians	Age-standardised Indigenous Australians	Other Australians
**Total population**	WA/SA^b^	Population	81,700	81,700	3,167,603
	WA/SA	Notifications	517	483	12,516
	WA/SA	Hospitalisations	148	172	893
	WA/SA/NT/QLD^c^	Population	246,189	246,189	6,766,712
	WA/SA/NT/QLD	Hospitalisations	490	569	1675
	All Australia	Population	429,261	429,261	17,677,150
	All Australia	ICU admissions	72	84	367
	All Australia	Deaths	22	33	157
**Chronic lower respiratory conditions**	WA/SA	Population^d^	14,663 (13,235–16,226)	17,137 (15,613–18,783)	439,754 (408,304–473,240)
	WA/SA	Notifications	65	83	763
	WA/SA	Hospitalisations	44	60	319
	WA/SA/NT/QLD	Population^d^	37,322 (34,639–40,178)	44,779 (41,901–47,835)	937,016 (887,116–989,293)
	WA/SA/NT/QLD	Hospitalisations	153	205	377
	WA/SA/NT/QLD	ICU admissions	31	36	62
	WA/SA/NT/QLD	Deaths	5	8	20
**Diabetes mellitus**	WA/SA	Population^d^	9283 (8113–10,588)	15,408 (14,093–17,157)	195,822 (174,218–219,831)
	WA/SA	Notifications	61	88	199
	WA/SA	Hospitalisations	37	58	97
	WA/SA/NT/QLD	Population^d^	25,984 (23,708–28,459)	38,877 (36,141–41,778)	374,113 (341,719–409,386)
	WA/SA/NT/QLD	Hospitalisations	135	201	114
	WA/SA/NT/QLD	ICU admissions	20	31	22
	WA/SA/NT/QLD	Deaths	4	8	3
**Obesity**^**e**^	WA/SA	Population^d^	4368 (3540–5372)	5562 (4625–6667)	83,783 (69,172–101,646)
	WA/SA	Notifications	35	35	162
	WA/SA	Hospitalisations	24	24	84
	WA/SA/NT/QLD	Population^d^	12,235 (10,630–14,054)	15,526 (13,730–17,546)	206,383 (180,718–235,327)
	WA/SA/NT/QLD	Hospitalisations	38	39	101
	WA/SA/NT/QLD	ICU admissions	7	8	30
	WA/SA/NT/QLD	Deaths	2	3	7
**Renal disease**	WA/SA	Population^d^	1515 (1070–2141)	2281 (1748–3047)	32,774 (24,391–43,713)
	WA/SA	Notifications	18	29	47
	WA/SA	Hospitalisations	13	21	39
	WA/SA/NT/QLD	Population^d^	4332 (3422–5465)	6707 (5736–8297)	70,243 (56,840–87,291)
	WA/SA/NT/QLD	Hospitalisations	72	108	42
	WA/SA/NT/QLD	ICU admissions	12	18	9
	WA/SA/NT/QLD	Deaths	2	4	2
**Cardiac disease**	WA/SA	Population^d^	4978 (4126–5997)	8283 (7247–9616)	165,974 (150,461–193,224)
	WA/SA	Notifications	40	67	144
	WA/SA	Hospitalisations	27	47	100
	WA/SA/NT/QLD	Population^d^	12,202 (10,635–13,984)	18,170 (16,667–20,754)	331,543 (297,059–360,666)
	WA/SA/NT/QLD	Hospitalisations	82	125	115
	WA/SA/NT/QLD	ICU admissions	18	24	22
	WA/SA/NT/QLD	Deaths	4	7	4

^a^ ≥ 15 years of age

^b^Western Australia / South Australia

^c^Western Australia / South Australia / the Northern Territory / Queensland

^d^Number of cases of chronic condition ≥ years of age calculated from health survey data and ABS census population data (with 95% confidence intervals)

^e^Data only available from > 25 years onwards

Rates of influenza were higher in the Indigenous compared to the non-Indigenous Australian total (with or without chronic disease) populations for notifications (WA/SA), hospitalisations (WA/SA and WA/SA/NT/QLD), ICU admissions (all Australia), and deaths (all Australia) (Table 5). The rate ratios (RR) for Indigenous compared to non-Indigenous were the same or higher in all chronic disease sub-populations compared to the RRs in the total population for the same level of severity (i.e. notifications, hospitalisations, ICU admissions and deaths).

**Table 5:** Indigenous: Non-Indigenous relative risk of influenza incidence amongst the total (general) population and chronic disease sub-populations^a^

	State/territory	Notifications— relative risk	Notifications— 95% CI^**b**^	Hospitalisations— relative risk	Hospitalisations— 95% CI	ICU admissions — relative risk	ICU admissions — 95% CI	Deaths — relative risk	Deaths — 95% CI
**All Australia**	WA/SA^c^	1·50	1·37–1·64	7·48	6·35–8·80	··	··	··	··
	WA/SA/NT/QLD^d^	··	··	9·34	8·50–10·27	··	··	··	··
	All Australia	··	··	··	··	9·46	7·47–11·99	8·71	5·99–12·67
**Chronic lower respiratory conditions**	WA/SA	3·26	2·60–4·09	5·62	4·26–7·40	··	··	··	··
	WA/SA/NT/QLD	··	··	13·63	11·50–16·15	14·63	9·71–22·05	10·13	4·47–22·94
**Diabetes mellitus**	WA/SA	9·37	7·30–12·03	12·68	9·17–17·54	··	··	··	··
	WA/SA/NT/QLD	··	··	25·39	20·19–31·94	34·88	20·23–60·15	68·40	18·30–255·55
**Obesity**	WA/SA	4·14	2·88–5·96	5·55	3·49–8·63	··	··	··	··
	WA/SA/NT/QLD	··	··	6·55	4·53–9·46	4·50	2·07–9·82	7·19	1·85–27·86
**Renal disease**	WA/SA	13·26	8·37–21·03	11·56	6·81–19·62	··	··	··	··
	WA/SA/NT/QLD	··	··	41·51	29·09–59·22	32·11	12·42–71·53	29·10	5·16–163·88
**Cardiac disease**	WA/SA	15·46	11·85–20·64	15·82	11·21–22·33	··	··	··	··
	WA/SA/NT/QLD	··	··	29·52	22·93–38·01	30·16	16·96–53·64	48·71	14·34–165·48

^a^Age-standardised

^b^Confidence interval

^c^Western Australia / South Australia

^d^Western Australia / South Australia / the Northern Territory / Queensland

For influenza notifications, the RR for Indigenous compared to non-Indigenous Australians were statistically significantly higher in all chronic disease populations than in the total population of all Australians (i.e. with or without chronic disease) (Table 5). For influenza hospitalisations in WA/SA, the RR for Indigenous compared to non-Indigenous point estimates were higher (confidence intervals did not overlap) amongst those with diabetes mellitus and cardiac disease; and not statistically different in those with chronic lower respiratory conditions, obesity, and renal disease, compared to the total population. However, in the larger geographic region of WA/SA/NT/QLD, point estimates for influenza hospitalisation were significantly higher in all chronic disease populations compared to the total population except for those with obesity, in whom there was no statistical difference.

For intensive care unit admissions, RR for Indigenous compared to non-Indigenous point estimates were higher for populations with diabetes mellitus, renal disease, and cardiac disease compared to the RR point estimates for the total population; and no significant difference was noted amongst those with chronic lower respiratory disease and obesity. For influenza deaths, RR for Indigenous compared to non-Indigenous were higher in those with diabetes mellitus and cardiac disease compared to the total population. The point estimates were higher but not statistically significant different in RR for Indigenous compared to non-Indigenous for the other chronic conditions (Table 5).

### Rate ratios for indigenous compared to non-indigenous, by severity of influenza disease

In the total population, RR for Indigenous compared to non-Indigenous were significantly higher for influenza hospitalisations compared to notifications (Table 5). However, there was no difference between RRs for ICU admissions and deaths.

In contrast to the total population, amongst the total chronic disease subgroups, there was no statistically significant difference between RRs for different levels of severity in any chronic disease population or geographic grouping, with one exception—a higher RR for hospitalisation compared to notification in those with chronic lower respiratory conditions in WA/SA.

## Discussion

As expected, we demonstrated higher rates of all five chronic conditions as well as higher rates of pandemic influenza notifications, hospitalisations, ICU admissions, and deaths amongst Indigenous Australians compared to non-Indigenous Australians. When the analysis was restricted to those with chronic diseases, we found that rate ratios for Indigenous compared to non-Indigenous for influenza disease, morbidity, and mortality were not lower than for the total population but, in the majority of instances, were higher than those for the total population. The increases were most marked amongst notifications, where the RRs were significantly higher in every chronic disease population than in the total population. However, for more severe outcomes, the differences in RRs between chronic disease subgroups and the total population (Table 5) were less consistent, and there was no clear trend of RRs with  within any population. This suggests that 1) the higher rates of influenza infection (notifications) amongst Indigenous Australians is not explained by higher rates of the five chronic disease categories we examined, and 2) a substantial proportion of the higher rates of more severe outcomes (hospitalisations, ICU admissions, and death) in Indigenous people with chronic conditions is a result of higher infection (notification) rates. Caution in interpretation of the more severe outcomes may be warranted, due to the wide confidence intervals for some of the rate ratios for whom case numbers were small, especially for more severe outcomes.

Our findings of higher disease rates in Indigenous Australians concurred with other data sources, which reported 8-fold hospitalisation and 6-fold death rates for influenza amongst Indigenous Australians compared to non-Indigenous Australians during the 2009 ‘swine flu’ pandemic [28]. Other researchers have also hypothesised that these disparities are likely to be attributable to a higher prevalence of chronic disease [2, 5]. Goggin et al. found that Indigenous status was not independently associated with hospitalisation in WA, after adjustment for age and the presence of two or more chronic diseases amongst those already diagnosed with H1N1 infection [16]. This is consistent with our finding of an increased risk of influenza infection amongst Indigenous compared to non-Indigenous people with chronic disease, which does not increase further for more severe outcomes. In contrast, Zarychanski et al. found that Indigenous status was independently associated with ICU admission for Canada’s First Nations in Manitoba after adjustment for comorbidity, age, sex, income, and rural location [3].

If higher rates of influenza notifications amongst Indigenous people are not explained by higher rates of chronic disease, what alternative explanations may there be? More vigilant testing of Indigenous people in Aboriginal Medical Services is possible. However, the majority of testing was done whilst there was a national universal testing policy for the first few months of the pandemic. Differential testing would have been more likely to influence notification rates than in those hospitalised with influenza. During the 2009 influenza pandemic within the Top End of Australia, encompassing the north part of the Northern Territory, universal testing was implemented and yielded a statistically significantly higher notification rate among Indigenous Australians as compared to non-Indigenous Australians, indicating ascertainment bias is not solely responsible [29]. A non-age-standardised serological survey from the Top End revealed an attack rate of 1·85-fold higher for Indigenous Australians than for non-Indigenous Australians [30]. Our age-standardised rates yielded a lower relative risk for influenza notifications (RR 1·50, 95%, CI 1·37–1·64); this may be explicable by pre-existing immunity in older age groups [31]. Non-clinical reasons for hospitalisation (e.g., residence in remote areas requiring admission for observation) are possible, but are unlikely to have any impact on laboratory diagnosis, ICU admission, or death. Certain clinical factors may have been relevant in the disparity in influenza notification rates. Chronic diseases of greater severity in Indigenous Australians may result in more cases of symptomatic influenza amongt those with these conditions, as may a greater burden of multiple chronic comorbidities, such as the concurrence of diabetes mellitus and renal disease [32]. Certain socioeconomic factors, such as a larger family size, overcrowding, and poorer access to sanitation facilities may increase influenza transmission within some Indigenous communities, and thus account for some of the disparity [33].

Genetic factors are unlikely to be relevant given the similar outcomes amongst genetically diverse Indigenous populations in Australia, New Zealand, and North America. Thus, there are factors relating to colonisation itself that appear to be partially causal in the gap between Indigenous and non-Indigenous populations [1–7, 34–36].

Physical health is also influenced by the downstream effects of intergenerational psychosocial trauma resulting from colonisation, loss of language and culture, and ongoing systematic, structural, and personal racism [11]. This results partly from the breakdown of traditional lifestyles and poor access to their modern replacements, such as 24-h health care, running water, and sanitation facilities. It also results from imposition of drastic environmental changes to which human physiology is maladapted (e.g., drastic changes in dietary quality and caloric volume) [10, 34]. Differences in health care access based on race have been demonstrated: once admitted to hospital for cardiovascular disease, Indigenous Australians received fewer medical interventions and prescriptions, but had poorer outcomes, than non-Indigenous Australians [11]. The Australian Human Rights Commission identifies financial poverty, chronic stress, lack of control over health services, lack of ownership of traditional lands, and social and political racism as social determinants of poorer health amongst Indigenous Australians [37]. A 2014 report stated that connection to traditional ways of life and living in one’s ancestral homelands, rather than educational attainment and monetary resources, was protective against chronic disease amongst Indigenous Australians [38]. Amongst Canadian First Nations communities, prevalence of suicide varied greatly based on presence of protective factors: self-government (most protective); successful land claims; community control over health and adjacent services; and presence of cultural facilities [39]. This all suggests that broader models of health, which address social inequalities, integrate Indigenous health knowledge into clinical practice, and in which collaboration and empowerment of community leaders occurs, may more effectively address the gap in severe influenza between Indigenous and non-Indigenous Australians [11, 15, 40]. This knowledge adds impetus to the Australian federal government initiative of ‘Closing the Gap’ in health outcomes between Indigenous and non-Indigenous Australians [41].

Our study had certain limitations. Self-reported data on chronic conditions from the ABS Health Surveys may not have perfectly matched the ascertainment of chronic conditions amongst influenza cases, this may or may not have affected rate ratios depending on whether the error was equal amongst Indigenous and non-Indigenous groups. There were small numbers of ICU admissions and deaths from influenza, causing sensitivity to small errors leading to wide confidence intervals and thus a lack of power to detect real differences. Rate ratios with confidence intervals are a conservative estimate of significance and may have underestimated significant differences in RR for Indigenous compared to non-Indigenous cases of influenza. Collection of complete Indigenous status data is a recognised challenge leading to data loss, as close to a third of cases of swine flu needing to be excluded, the direction of change on RR for Indigenous compared to non-Indigenous is uncertain. Data on influenza amongst those with multiple comorbidities and those with no chronic disease were not available, thus the RR amongst all Australia may have been higher and the contrast to those with specific chronic conditions lower, than if we were able to calculate RR amongst Australians without chronic conditions.

## Conclusions

We are the first investigators to access and analyse data from a large geographic area on chronic disease prevalence and its relationship to pandemic influenza incidence and severity amongst Indigenous people. Amongst those with chronic diseases, Indigenous Australians had significantly higher rates of influenza infection than non-Indigenous Australians with chronic disease; however, there was no further incremental increase in rates of severe influenza. Thus, higher background prevalence of chronic disease in Indigenous Australians does not account for the overall higher rates of 2009 H1N1 influenza pandemic (influenza A(H1N1)pdm09) in Indigenous Australians, but may contribute to more severe outcomes. Broader social and cultural factors also may play an important role.

## Supplementary Information

**Additional file 1.** Diseases as grouped in health survey analyses.

## Data Availability

The data that support the findings of this study were obtained upon author application to various sources. 1. Prevalence data for chronic conditions were obtained by analysing the Confidentialised Unit Record Files of health surveys conducted by the Australian Bureau of Statistics (ABS). 2. We obtained data on influenza cases during the 2009 ‘swine flu’ pandemic from the Communicable Diseases Network of Australia (CDNA), a division of the Department of Health and Aging (DoHA). Restrictions apply to the availability of both these sets of data, which were used under license for the current study, and so are not publicly available. Data are, however, available from the authors upon reasonable request and with written permission of Australian Bureau of Statistics and Department of Health. 3. Percentage prevalence of each condition in the morbidity, mortality, and health survey data were adjusted using weightings for age, gender, probability of being selected, non-response rates and state or territory provided to the ABS to adjust for differences between survey and census populations: this was obtained by accessing the 2011 Australian census available at Commonwealth of Australia, Australian Bureau of Statistics, available from https://www.abs.gov.au/AUSSTATS/abs@.nsf/allprimarymainfeatures/E8D5DE6EDB7BB6A4CA2582F90013E6A1.
