# Supplementary material for: The role of chronic disease in the disparity of influenza incidence and severity between indigenous and non-indigenous Australian peoples during the 2009 influenza pandemic
Source: BMC Public Health. 2022 Jul 5;22:1295. doi: 10.1186/s12889-022-12841-6 (PMC9254512; doi:10.1186/s12889-022-12841-6)
Supplement: Supplementary file 1 — Additional file 1. Diseases as grouped in health survey analyses. [file 12889_2022_12841_MOESM1_ESM.docx]

**The role of chronic disease in the disparity of influenza incidence and severity between Indigenous and non-Indigenous Australian Peoples during the 2009 influenza pandemic**

**Dixit, Rashmi; Webster, Fleur; Booy, Robert; Menzies, Robert**

*Corresponding Author*

**Dixit, Rashmi, PhD**^1^

c/o Westmead Clinical School

University of Sydney

NSW 2145

Australia

tropid7@gmail.com

+61448886199

**Webster, Fleur, MPH**^2^

c/o School of Public Health and Community Medicine

University of New South Wales

NSW 2052

Australia

fleur_webster@hotmail.com

**Booy, Robert, MD**^1,3^

c/o Westmead Institute for Medical Research

NSW 2145

Australia

robert.booy@sydney.edu.au

**Menzies, Robert, PhD**^2^

c/o Kirby Institute, Faculty of Medicine

University of New South Wales

NSW 2052

Australia

rob_menzies60@hotmail.com

**Correspondence to: Dixit, Rashmi (tropid7@gmail.com)**

1. University of Sydney

2. University of New South Wales

­­3. Westmead Institute for Medical Research

**Additional file 1: Diseases as grouped in health survey analyses**

***cardiac disease***

current or long-term ischaemic heart disease, angina, heart attack, heart failure, other heart diseases

***chronic lower respiratory conditions***

current or long-term bronchitis, asthma, emphysema

***diabetes mellitus*** *(excluding gestational diabetes)*

type 1 diabetes mellitus, type 2 diabetes mellitus, type unknown diabetes mellitus, high blood or urine glucose

***obesity***

measures BMI > 40

***renal disease***

chronic, long-term renal disease defined by reduction in glomerular filtration rate
